# Gephyrin phosphorylation in the functional organization and plasticity of GABAergic synapses

**DOI:** 10.3389/fncel.2014.00103

**Published:** 2014-04-09

**Authors:** Paola Zacchi, Roberta Antonelli, Enrico Cherubini

**Affiliations:** ^1^Department of Neurosciences, Scuola Internazionale Superiore di Studi AvanzatiTrieste, Italy; ^2^European Brain Research InstituteRoma, Italy

**Keywords:** gephyrin, phosphorylation, GABA_A_ receptors, GSK-3β signaling, ERK signaling, Pin1

## Abstract

Gephyrin is a multifunctional scaffold protein essential for accumulation of inhibitory glycine and GABA_A_ receptors at post-synaptic sites. The molecular events involved in gephyrin-dependent GABA_A_ receptor clustering are still unclear. Evidence has been recently provided that gephyrin phosphorylation plays a key role in these processes. Gephyrin post-translational modifications have been shown to influence the structural remodeling of GABAergic synapses and synaptic plasticity by acting on post-synaptic scaffolding properties as well as stability. In addition, gephyrin phosphorylation and the subsequent phosphorylation-dependent recruitment of the chaperone molecule Pin1 provide a mechanism for the regulation of GABAergic signaling. Extensively characterized as pivotal enzyme controlling cell proliferation and differentiation, the prolyl-isomerase activity of Pin1 has been shown to regulate protein synthesis necessary to sustain the late phase of long-term potentiation at excitatory synapses, which suggests its involvement at synaptic sites. In this review we summarize the current state of knowledge of the signaling pathways responsible for gephyrin post-translational modifications. We will also outline future lines of research that might contribute to a better understanding of molecular mechanisms by which gephyrin regulates synaptic plasticity at GABAergic synapses.

## Introduction

Post-synaptic scaffolding molecules are key factors for the functional organization of synapses. They ensure the accurate accumulation of neurotransmitter receptors in precise apposition to pre-synaptic release sites as required for a reliable synaptic transmission. Scaffolding molecules also interact with cytoskeletal anchoring elements and these interactions are thought not only to provide the physical constraints for maintaining receptors at synapses, but also for regulating the constant flux of receptors and scaffolding elements in and out of post-synaptic sites (Choquet and Triller, 2003; Hanus et al., 2006). They can also regulate downstream signaling pathways to adjust the molecular composition of the post-synaptic devices necessary to sustain synaptic plasticity. At inhibitory post-synaptic densities (PSDs) a single protein, gephyrin, builds the major scaffold for the transient immobilization of inhibitory glycine receptors (GlyRs) and α2-γ2 subunits containing GABA_A_ receptors (GABA_A_Rs; Tretter et al., 2012). The formation and maintenance of gephyrin clusters rely mostly on gephyrin-gephyrin interactions (reviewed in Fritschy et al., 2008). Gephyrin is a 93-kDa protein that consists of three major domains: an N-terminal G-domain, a C-terminal E-domain and a connecting central linker region (C-domain) (Prior et al., 1992). Crystal structure studies have demonstrated that while the G-domain has an intrinsic tendency to trimerize the E-domain dimerizes (Schwarz et al., 2001; Sola et al., 2001, 2004). These oligomerization features suggest a model for cluster formation whereby gephyrin builds a bidimensional hexagonal lattice underneath the synaptic membrane (Kneussel and Betz, 2000; Schwarz et al., 2001; Sola et al., 2001, 2004; Xiang et al., 2001) which exposes a high number of binding sites for GlyR β subunits and for GABA_A_Rs α1, α2, α3, β2 and β3 subunits (Maric et al., 2011; Kowalczyk et al., 2013).

Recently, an elegant study based on quantitative three-dimensional nanoscopic imaging, has not only confirmed that gephyrin clusters are indeed bidimensional planar structures lying underneath the synaptic plasma membrane but has also provided evidence that all gephyrin molecules in the cluster are potentially capable to interact with neurotransmitter receptors localized in the synaptic membrane in a stoichiometry ratio gephyrin-receptor of approximately 1:1 (Specht et al., 2013).

A consequence of this organization is that changes in gephyrin clustering could produce parallel changes in the number of receptors trapped by the scaffold, and thus lead to corresponding alteration of the strength of synaptic transmission. This may vary with age and in different cell compartments as suggested by the transient expression of gephyrin clusters co-localized with GABA_A_Rs at immature perisomatic but not dendritic basket-Purkinje cell synapses (Viltono et al., 2008). The loss of gephyrin and the consequent re-organization of perisomatic GABA_A_R clusters in more mature neurons may affect their trafficking and stability. Another important element in the functional organization of inhibitory synapses is represented by the affinity of gephyrin for neurotransmitter receptors (Fritschy et al., 2008). Mechanisms that are able to alter these parameters could uncouple gephyrin clustering and the number of receptors that can be effectively accommodated within the cluster itself. This mechanism would be well suited for the complex and still poorly understood dynamics of gephyrin-dependent GABA_A_Rs (Tretter et al., 2012). In contrast to GlyRs that interact with gephyrin only through the β subunits, GABA_A_Rs interact *via* their large intracellular loops with several subunits of the α and β families such α1, α2, α3 and β2, β3, respectively (Tretter et al., 2008, 2011; Saiepour et al., 2010; Mukherjee et al., 2011; Kowalczyk et al., 2013). These subunits utilize the same binding site as GlyR (Maric et al., 2011) but display a binding affinity at least one order of magnitude lower. The γ2 subunit, initially thought to be implicated in controlling gephyrin-dependent GABA_A_Rs clustering (Essrich et al., 1998), as its gene deletion strongly affects both receptor and gephyrin synaptic accumutation (Günther et al., 1995), was never identified as direct interactor of gephyrin (Tretter et al., 2012). The α4, α5 and δ subunits present mainly on extrasynaptic GABA_A_Rs lack of co-localization with gephyrin (Farrant and Nusser, 2005). While each GABA_A_R is a pentamer, it is still not known which available binding sites are actively involved in gephyrin interaction and whether and how they cooperate to increase the overall binding affinity for gephyrin. Finally, gephyrin dynamics rely on its availability for cluster formation which depends on its regulated transport to post-synaptic sites and degradation. Degradation requires mainly the activity of the Ca^2+^-dependent cysteine protease calpain-1 (Kawasaki et al., 1997; Tyagarajan et al., 2011).

The recruitment of gephyrin to GABAergic synapses needs the contribution of at least two classes of interactors: the cell adhesion molecules of the neuroligin (NL) family (Südhof, 2008) and the guanine nucleotide exchange factor for the monomeric GTPase Cdc42 collybistin (Kins et al., 2000). In particular NL2, the isoform constitutively localized at inhibitory GABAergic synapses (Varoqueaux et al., 2004), interacts with both gephyrin and collybistin forming a ternary complex able to activate collybistin-driven gephyrin tethering to the plasma membrane followed by receptors recruitment (Poulopoulos et al., 2009).

In summary, several gephyrin-dependent mechanisms affect the number of GABA_A_Rs at synaptic sites at any given time, and thereby may influence the strength of synaptic transmission: gephyrin-gephyrin interaction, gephyrin-receptor (neurotransmitters or other synaptically localized membrane proteins) binding affinities, gephyrin turnover and synaptic transport. Recently new mechanistic insights on the regulation of gephyrin oligomerization, stability and receptor binding capability have been provided. They suggest that phosphorylation, (a versatile mechanism for regulating protein activity in a specific and controlled manner), already involved in the functional modulation of receptors at synapses, is determinant for all aspect of gephyrin dynamics. Interestingly, the signaling pathways altering the phosphorylation status of gephyrin have been previously identified as modulator of glutamatergic signaling. The functional cross-talk between excitatory and inhibitory transmission may have important implications for the long-term stability of neuronal networks.

## Signaling pathways involved in gephyrin clustering

**Figure 1:**
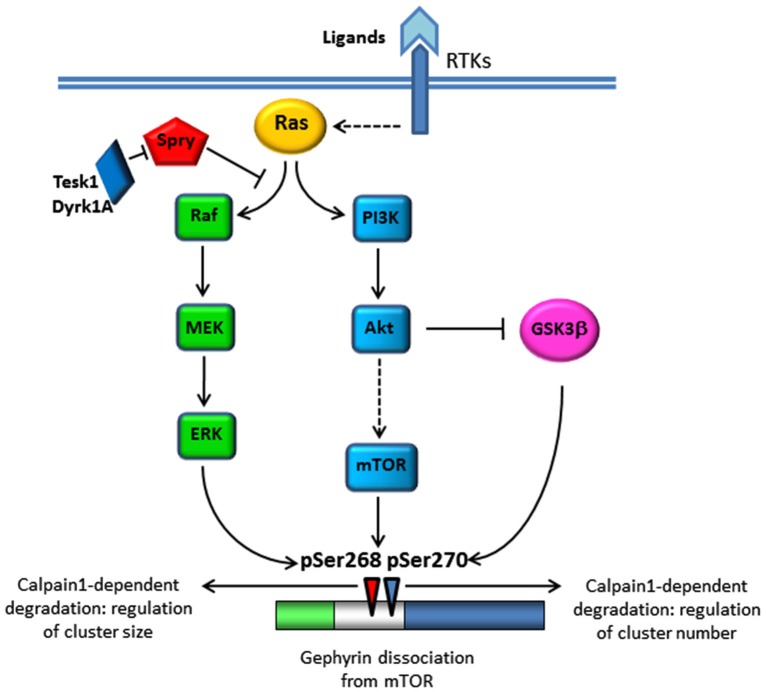
**Schematic representation of signaling pathways affecting gephyrin clustering**. Stimulation of RTKs by ligand binding or activity-dependent increase in calcium levels activates Ras and its downstream signaling cascades Ras/MAPK and PI3K/Akt leading to gephyrin phosphorylation at Ser268 by ERK and gephyrin dissociation from mTOR. Akt also inhibits GSK-3β activity, the kinase responsible of Ser270 phosphorylation. Gephyrin phosphorylated by these two kinases becomes substrate of calcium-dependent calpain degradation.

A recent genome-wide siRNA screening aimed at identifying protein kinases stabilizing gephyrin clustering revealed a contribution of Receptor Tyrosine Kinases (RTKs) signaling; in particular the tropomyosin-related kinase B (Trk-B) and its ligand the brain-derived neurotrophic factor (BDNF; Wuchter et al., 2012). The BDNF-TrkB system is required for multiple aspects of neuronal functions including neuronal survival and differentiation during development as well as synaptic plasticity of mature neurons (Thoenen et al., 1987; Tanaka et al., 2000; Poo, 2001). The activation of TrkB by BDNF triggers various signaling cascades including the Ras/mitogen-activated protein (MAP) kinase (Ras/MAPK) pathway, the phosphatidylinositol 3-kinase (PI3-Kinase)/Akt pathway and the phospholipase C gamma (PLCγ) pathway (Arévalo and Wu, 2006). At glutamatergic synapses, the activation of MAPK and PI3K pathways plays a crucial role in synaptic plasticity. This occurs not only *via* *de novo* regulation of protein synthesis but also *via* trafficking of pre-existing synaptic proteins. Therefore, it is not surprising that these signaling pathways contribute to regulate gephyrin transport at synapses (Figure 1). The BDNF-dependent activation of the PI3K/Akt pathway leads to the activation of rapamycin (mTOR), a regulator of mRNA translation (Sarbassov et al., 2005). Sabatini et al. (1999) demonstrated that mTOR interacts with gephyrin and this interaction is fundamental for mTOR-dependent signaling to the translational repressor 4E-BP1 (Sabatini et al., 1999). Upon BDNF treatment mTOR decreases its association with gephyrin, thus releasing gephyrin for membrane transport and cluster assembly. In addition PI3K activation, by promoting an increase in phosphatidylinositol (3,4,5)-triphosphate (PIP3) membrane content, may enhance collybistin-mediated gephyrin recruitment at GABAergic synapses (Reddy-Alla et al., 2010). In parallel, BDNF-dependent activation of Akt was shown to promote the inactivation of the serine/threonine kinase glycogen synthase kinase 3β (GSK-3β), a recently indentified negative regulator of gephyrin clustering (Tyagarajan et al., 2011). The authors of the wide-genome screening (Wuchter et al., 2012) also provided evidence for a contribution of the MAPK signaling cascade to gephyrin clustering, independent of mTOR activation, and controlled by the negative regulators of RTKs signaling sprouty proteins (Kim and Bar-Sagi, 2004).

The screening identified two siRNA directed against testicular protein kinase 1 (Tesk1) and Dual specificity tyrosine-phosphorylation-regulated kinase 1A (Dyrk1A), two protein kinases implicated in the inhibitory phosphorylation of sprouty proteins, in particular sprouty2, that specifically inhibit the Ras-Raf-MAPK pathway triggered by BDNF (Aranda et al., 2008; Chandramouli et al., 2008). This study, while revealing mechanisms involved in the control of gephyrin clustering, did not address the possibility that such signaling cascade may also affect gephyrin phosphorylation. Tyagarajan et al. (2013) were able to demonstrate that some of the kinases belonging to the MAPK and PI3K/Akt signaling pathways influence gephyrin dynamics and GABAergic transmission right through direct gephyrin phosphorylation (see below).

## Phosphorylation of gephyrin C-domain alters its oligomerization and stability properties

Gephyrin has been known to be a phosphoprotein since 1992, when Langosh and colleagues discovered that this protein co-purified with GlyR preparations has a kinase activity capable of promoting the incorporation of phosphate groups into serine and threonine residues (Langosch et al., 1992). The functional relevance of these post-translational modifications was neglected for long time, possibly because gephyrin was considered to be just a mere tubulin-binding protein, therefore a simple structural component of the inhibitory PSD.

**Figure 2:**
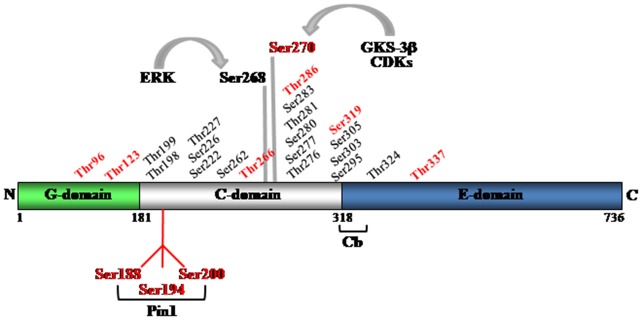
**Schematic representation of gephyrin domains and the identified phosphorylation sites**. Mass spectrometry has allowed identifying 22 serine and threonine residues within the C-domain and one (threonine 324), in the E-domain. In red are highlighted all putative Pin1 consensus motifs. Ser270 and Ser268 are recognized targets of GSK-3β and ERK kinase activities, respectively.

Mass spectrometry analysis performed on gephyrin isolated from either mouse or rat brain homogenates or purified upon its overexpression in eukaryotic cells, has identified 22 phosphorylation sites, all located within the C-domain of gephyrin, except the threonine 324 (Thr324) site that lies in the C-terminal E-domain (Figure 2; Herweg and Schwarz, 2012; Kuhse et al., 2012; Tyagarajan et al., 2013). The C-domain is positioned between the highly conserved G- and E-domains that are directly involved in gephyrin multimerization. Based on its sensitivity to proteolytic cleavage (Schrader et al., 2004), the C-domain is the most exposed to the surrounding environment, making it a suitable substrate for post-traslational modifications. This domain also mediates the phosphorylation-dependent recruitment of the peptidyl prolyl *cis-trans* isomerase Pin1 (discussed below) (Zita et al., 2007), the interaction with dynein light chain (Fuhrmann et al., 2002) and contributes to the recruitment of collybistin (Zacchi et al., personal communication).

In this region, conformational changes induced by phosphorylation could affect the folding of the C-domain itself and of the neighboring G- and E-domains, thus altering gephyrin clustering properties. A recent study (Herweg and Schwarz, 2012) has demonstrated that gephyrin, once expressed in a system that allows post-translational modifications, behaves quite differently in terms of oligomerization, folding stability and receptor binding. Gephryn expressed in Spodoptera frugiperda (Sf9) insect cells shows a diffuse distribution in the cytosol instead of the characteristic “aggregates” observed in HEK293 (Meier et al., 2000) or COS7 cells (Kirsch and Betz, 1995). The basic building blocks are formed by hexamers instead of trimers; in addition, G- and C-domains form a complex with increased overall stability while E-domains are stabilized upon receptor interaction. These parameters are also sensitive to changes in the amino acid sequence of gephyrin due to alternative splicing of the gene that, interestingly, impacts mostly on its C-domain organization, further underlying the contribution of this region in determining gephyrin folding and clustering (Herweg and Schwarz, 2012). It is therefore not surprising that most of the signaling pathways able to affect gephyrin clustering are represented by serine/threonine kinases targeting specific residues embedded in the C-domain of the protein.

## Phosphorylation of gephyrin at serine 270 is at the cross-road of different signaling pathways

One of the first gephyrin residues identified as the target of specific kinases was serine 270 (Ser270; Tyagarajan et al., 2011). Interestingly, the first kinase found to promote post-translational modifications was a serine/threonine kinase belonging to the family of Glycogen Synthase Kinase 3 (GSK3), enzymes originally identified as key regulators of glucose metabolism (Woodgett and Cohen, 1984; Wang and Roach, 1993). GSK3 signaling cascades have clearly recognized roles in neurodevelopmental processes such as neurogenesis, neuronal migration, neuronal polarization and axonal growth and guidance (reviewed in Hur and Zhou, 2010). Recently they have been implicated in N-methyl-D-aspartate receptors (NMDARs)-dependent long-term depression at glutamatergic synapses (Bradley et al., 2012). Even though the underlying molecular mechanisms are still not understood, GSK-3β–dependent phosphorylation of PSD-95, the major scaffold protein of excitatory PSD, functionally homologue of gephyrin, was found to destabilize the scaffold molecule thus allowing AMPA receptors internalization and LTD induction (Nelson et al., 2013).

Like PSD-95, GSK-3β appears to exert a negative effect on gephyrin clustering at GABAergic synapses (Tyagarajan et al., 2011). Several lines of evidence support this notion. Overexpression of a gephyrin phosphodeficient mutant (Ser270Ala) in cultured hippocampal neurons promotes the formation of supernumerary gephyrin clusters similar in size to those obtained upon wild-type gephyrin overexpression. Functionally, alanine mutation at this site selectively enhances the frequency of miniature inhibitory post-synaptic currents (mIPSC), a result which is in line with the increased density of functional GABAergic synapses. Additionally, a similar phenotype was observed upon pharmacological inhibition of GSK-3β activity both *in vitro* and *in vivo*. The authors of this study also provided mechanistic insights on how GSK-3β dependent phosphorylation of Ser270 can negatively regulate gephyrin clustering. They were able to demonstrate that phosphorylated gephyrin becomes substrate of the Ca^2+^-dependent protease calpain-1, possibly because at this location the phosphorylation-dependent conformational change may expose the sequence rich in proline (P), glutamic acid (E), serine (S) and threonine (T) (PEST sequence; Rechsteiner, 1990) that acts as a signal peptide for protein degradation. It is interesting to note that Ser270 lies also within a putative Pin1 consensus motif, raising the intriguing possibility that prolyl-isomerase may also participate in the conformational changes required to drive gephyrin proteolytic degradation. Since rises in calcium and GSK-3β activation are coupled to neuronal activity, the identified mechanisms are well suited to mediate plasticity-related changes at GABAergic synapses. Several issues remain to be unraveled regarding the functional consequences of this phosphorylation event. It will be interesting to understand how Ser270 phosphorylation destabilizes gephyrin assembled into a crowded lattice, where gephyrin is engaged in several protein-protein interactions with itself, neurotransmitter receptors and other transmembrane proteins (e.g., NL2). All these interactions represent potential targets of the signaling cascade. The fact that gephyrin phosphodeficient mutants possess synaptogenic activity further supports the notion that this site may regulate gephyrin binding to proteins important for building and maintaining functional GABAergic synapses. By converging on both scaffold molecules PSD-95 and gephyrin, GSK-3β signaling cascade, coordinates changes at both glutamatergic and GABAergic synapses, thus allowing to maintain an appropriate excitatory/inhibitory (E/I) balance.

The picture became even more complicated by the discovery that other kinases of the CDK family, in particular Cyclin-dependent kinase 5 (Cdk5), can target the same site, making this residue at the cross-road of different signaling pathways (Kuhse et al., 2012). Cdk5 is a proline-directed serine/threonine kinase with high activity in the central nervous system. Based on sequence homology, Cdk5 belongs to a class of kinases operating in the cell cycle, even though it is not activated by traditional cyclins and it plays critical roles in several aspect of brain development and neuronal functions including neuronal migration, differentiation, synapse development and plasticity (Lai and Ip, 2009; Su and Tsai, 2011).

The precise role of Cdk5 in activity-dependent synaptic plasticity is still not understood but the identification of novel substrates and interacting molecules has provided significant mechanistic insights. At glutamatergic synapses, Cdk5 has been shown to affect NMDA receptors-dependent plasticity through several mechanisms: (i) by altering NMDA receptor channel conductance upon Cdk5-depenent phosphorylation of certain receptor subunits (Li et al., 2001); (ii) by down-regulating in an activity-dependent manner NMDA receptors number *via* a calpain-dependent proteolytic degradation (Hawasli et al., 2007); and (iii) by regulating the endocytosis of NMDA receptor *via* phosphorylation of the scaffolding molecule PSD-95 (Morabito, 2004; Zhang et al., 2008).

Members of Cdk family, in particular Cdk5, contribute to gephyrin posphorylation at Ser270. Interestingly, this event seems to be tightly controlled by the level of expression of collybistin, being its down-regulation associated with a loss of gephyrin immunoreactivity as detected by the widely used monoclonal antibody mAb7a (Kuhse et al., 2012). The authors of this study showed that the antibody mAb7a is sensitive to gephyrin phosphorylation at that specific amino acid residue, making it a *bona fide* phospho-Ser270-specific monoclonal antibody. Therefore, the observed drastic reduction of mAb7a immunoreactivity observed upon collybistin knock-down or pharmacological inhibition of CDKs in cultured hippocampal neurons, indicated a reduction in gephyrin phosphorylation at Ser270 not necessarily associated with loss of synaptic gephyrin puncta. Experiments performed by using another gephyrin-specific antibody, not sensitive to its phosphorylation status, indeed demonstrated that the number and size of gephyrin clusters were not significantly affected by these treatments. Based on these results, in a mature cluster, gephyrin is expected to be constitutively phosphorylated at position 270, detectable by the mAb7a antibody, and to undergo selective dephosphorylation upon collybistin down-regulation. In contrast, results obtained from the characterization of GSK-3β dependent phosphorylation of gephyrin support an opposite scenario. Gephyrin assembled into a cluster is expected to be mainly dephosphorylated and to undergo activity-dependent GSK-3β mediated phosphorylation to promote its proteolytic degradation followed by cluster disassembly (Tyagarajan et al., 2011). Several speculations can be put forward to place these conflicting results in a more coherent picture. One possibility is that gephyrin builds different types of clusters, the one detected by mAb7a being characterized by high turnover rates. Alternatively, gephyrin scaffold is heterogenous in respect to gephyrin modifications and that phosphorylation at Ser270, as well as at neighboring positions, may generally act by restricting gephyrin oligomerization potential.

A question raised by these findings is how collybistin exerts its regulatory effect on Cdk5-dependent gephyrin phosphorylation. Collybistin is a key interactor of gephyrin known to participate in its membrane recruitment and synaptic targeting (Papadopoulos and Soykan, 2011). This activity relies on the presence of a Pleckstrin homology domain in collybistin sequence, a domain thought to mediate the attachment of the molecule to the membrane by binding to phosphoinositides (Hyvönen et al., 1995). Most collybistin isoforms expressed in neurons possesses at their N-terminus an SH3 regulatory domain that prevents their membrane-targeting function (Kins et al., 2000; Harvey et al., 2004). At GABAergic synapses only the cell adhesion molecule NL2 (Poulopoulos et al., 2009) and the α2 subunit of GABA_A_Rs (Saiepour et al., 2010) are capable of relieving such SH3-mediated inhibition, possibly by binding to it, thus promoting a controlled recruitment of gephyrin scaffold. The authors of this study did not investigate the molecular mechanism responsible for collybistin influence on Cdk5 activity. Since Cdk5-dependent phosphorylation of gephyrin is controlled by collybistin expression level, one possible explanation is that Cdk5 catalytic activity is under the control of collybistin because it interacts with it or because gephyrin, while interacting with collybistin, better exposes the side chain of the amino acid residue undergoing post-translational modification.

## ERK-dependent phosphorylation of gephyrin at Ser268 affects clusters size and density

Over the past decade, the ERK/MAPK (extracellular signal-regulated protein kinase/mitogen-activated protein kinase) pathway has been implicated in many forms of synaptic plasticity at glutamatergic synapses, including NMDA-dependent and independent forms of LTP. ERK1/2 activity enhances AMPA receptor functional properties by affecting their trafficking, by promoting the structural remodeling of activated spines as well as local protein synthesis (Thomas and Huganir, 2004). At GABAergic synapses ERK1, and to a lesser extent ERK2, were shown to be responsible for gephyrin phosphorylation at a serine residue located in close proximity to the previously recognized target of GSK-3β activity, namely serine 268 (Ser268). This residue attracted attention also because it is not phosphorylated in the C3-gephyrin splice variant, the isoform mainly expressed in non-neuronal cells (Ramming et al., 2000), and this suggests a selective biological significance in neurons.

ERK-mediated phosphorylation at this position was shown to specifically affect the size of post-synaptic gephyrin clusters. Interestingly, ERK and GSK-3β–catalyzed phosphorylations at their corresponding positions became to be functionally interconnected, leading to a coordinated regulation of cluster size and density paralleled by corresponding changes in amplitude and frequency of GABAergic mIPSCs (Tyagarajan et al., 2013). In other words by inhibiting ERK activity, both cluster density and size were affected, suggesting that ERK exerts a control over GSK-3β activity.l. While the precise dynamics of these events is still unknown it is worth noting that both sites are embedded in a gephyrin domain that contains phosphorylation residues, including putative targets of the prolyl-isomerase Pin1 activity (see below), which render the scenario more complex. Moreover, Ser268 was found acetylated (together with additional nine residues). Even though the functional significance of this type of post-translational modification is unknown, Tyagarajan et al. (2013) hypothesized that acetylation may prevent unwanted phosphorylation by ERK and subsequent down-regulation of GABAergic transmission. Interestingly, ERK activity enhances the strength of glutamateric transmission while decreasing GABAergic transmission, leading to a shift of the E/I balance toward excitation. Therefore, dephosphorylation at Ser268 and/or its acetylation may represent plausible mechanisms to counteract the action of ERK at inhibitory synapses.

Though several issues still remain to be solved, ERK-mediated phosphorylation regulates cluster size *via* calpain activity, as previously demonstrated for GSK-3β–dependent regulation of cluster density. It is interesting to note that application of a broad spectrum phosphatase inhibitor to cultured hippocampal neurons was able to promote the reduction in size of gephyrin clusters, further supporting the functional role of phosphorylation in calpain-dependent gephyrin degradation (Bausen et al., 2010).

## Pin1: a new player in the organization of inhibitory post-synaptic specializations

Protein phosphorylation on serine and threonine residues preceding a proline (the so-called proline-directed phosphorylation) has been shown to regulate cell signaling through conformational changes that are not simply due to the phosphorylation event *per se*. Peptidyl-prolyl isomerization of phosphorylated Ser/Thr-Pro sites represents the molecular mechanism utilized by Pro-directed phosphorylation to switch a target substrate between two different functional conformations. The existence of the mechanism relies on the unique stereochemistry of proline residues that within native polypeptides can adopt both *cis* and *trans* conformations. *Cis*-to-*trans* and *trans*-to-*cis* isomerization occur spontaneously but at very low rate: the speed of this event being further reduced upon serine or threonine phosphorylation (Yaffe et al., 1997). These conversions are greatly accelerated by ubiquitous enzymes named peptidyl-prolyl *cis-trans* isomerases (PPIases) or rotamase (Fanghänel and Fischer, 2004). These are divided into 4 families that are unrelated in their primary sequences and three-dimensional structures even though they catalyze the same reaction: cyclophilins (Cyps), FK506-binding proteins (FK506s), parvulins and the PP2A phosphatase activator (PTPA; Jordens et al., 2006). Pin1 and its homologs belong to the parvulin subfamily of PPIase and are the only known enzymes able to isomerise phosphorylated Ser/Thr-Pro sites that become resistant to the catalytic action of conventional prolyl-isomerases (Yaffe et al., 1997). This feature makes the action of Pin1 relevant in the modulation of signaling events, taking into account that Pro-directed kinases and phosphatases are conformation-specific and act only on the *trans* conformation (Weiwad et al., 2000; Zhou et al., 2000).

Pin1 was initially discovered by its ability to interact with the fungal mitotic kinase NIMA (Never In Mitosis A), pointing to an exclusive role for Pin1 in mitosis (Lu et al., 1996). The rapid identification of novel Pin1 substrates has clearly unveiled that this enzyme exerts control over a plethora of cellular processes not only in actively dividing cells but also in fully differentiated cells like post-mitotic neurons. Up to now the best characterized neuronal Pin1 substrates are represented by cytoskeletal proteins such as *tau*, amyloid-β-protein precursor, α-synuclein, and neurofilaments since aberrant interactions with these have implications for the development of neurodegenerative disorders such as Alzheimer disease (Lee et al., 2011), Parkinson disease and amyotrophic lateral sclerosis (Rudrabhatla and Pant, 2010). The involvement of Pin1 in physiological apoptotic events required for the proper development of the nervous system has been also identified (Becker and Bonni, 2006) as well as its contribution for long-lasting forms of synaptic plasticity at excitatory synapses (Westmark et al., 2010).

Gephyrin was identified as a novel target of post-phosphorylation prolyl-isomerization long before its identification as target of Ras/MAPK and PI3K/Akt signaling cascades (Zita et al., 2007). Based on a naïve approach, by inspecting gephyrin amino acid sequence, it was possible to identify 10 putative Pin1 consensus motifs mostly concentrated in the C-domain of gephyrin (Figure 2). In particular, while two clusters of three consensus sites were found to be localized within the C-domain, two additional couple of epitopes were located close to the C-terminus of the G-domain and close to the N-terminus of the E-domain, respectively. The C-domain’s cluster encompassing the proline-rich region of gephyrin and containing serine 188, 194 and 200, was shown to be responsible for Pin1 recruitment, thus allowing Pin1-driven conformational changes of gephyrin substrate. Functionally, such structural remodeling of gephyrin molecule was shown to affect its binding affinity for the β subunit of the GlyR without affecting its oligomerization properties. In agreement with these findings, hippocampal neurons derived from Pin1 knockout mice demonstrated a loss in the number of GlyR immunoreactive puncta which were mirrored by a concomitant reduction in the amplitude of glycine-evoked currents. These data demonstrated for the first time that post-phosphorylation regulatory mechanisms can affect gephyrin-dependent clustering of inhibitory receptors, rendering it a potential mechanism involved in remodeling the post-synaptic device to sustain synaptic plasticity.

**Figure 3:**
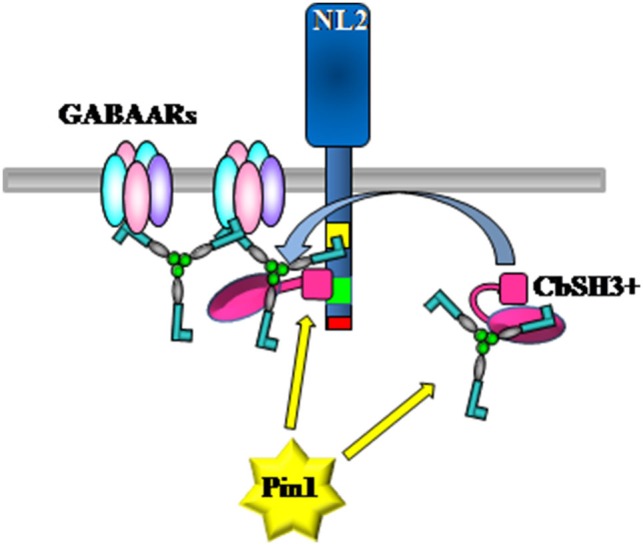
**Model of collybistin-driven recruitment of gephyrin by NL2 at GABAergic postsynapses**. Pin1 may affect gephyrin/collybistin as well as gephyrin/NL2 interactions leading to an increase or decrease in gephyrin deposition at post-synaptic sites. The cytoplasmic domain of NL2 contains a gephyrin binding domain (yellow), a putative CBD (green) and a C-terminal PDZ binding domain (red).

Is Pin1 also involved in GABAergic synaptic signaling? As already mentioned, gephyrin contribution to GABA_A_R dynamics requires the coordinated activity of several other associated proteins whose identification and functional characterization has just started to be addressed. At least two key molecules have emerged to play an essential role in regulating gephyrin accumulation at postsynapses, namely NL2 and collybistin (Poulopoulos et al., 2009). These molecules both possess in their sequences putative Pin1 consensus motifs, raising the intriguing possibility that post-phosphorylation prolyl-isomerization regulates their reciprocal interaction leading to changes in gephyrin dynamics at synaptic sites (Figure 3). Based on this notion, it will be interesting to characterize whether alanine mutagenesis of specific Pin1 consensus sites, in particular the one located within the domains actively engaged in the interaction, would alter (enhancing or weakening) their binding affinity. In addition, it has been demonstrated that, to interact with NL2, gephyrin utilizes a region encompassing the whole C-terminal E-domain linked to a portion of the central region (amino acid 286-736). Two Pin1 consensus sites are present within this gephyrin portion, namely Ser319 and Thr337. As described above, mass spectrometry analysis performed on gephyrin immunoprecipitated from whole rat brain lysates showed that at least Ser319 is phosphorylated *in vivo* (Tyagarajan et al., 2013), making it able to modulate gephyrin/NL2 interaction. In addition, Ser319-Pro is located at the C-terminus of a short amino acid sequence identified as the collybistin binding domain (CBD) on gephyrin (Harvey et al., 2004). Interestingly, the CBD also contains two crucial residues for the interaction with GABA_A_Rs α1, α2, and α3 subunits, namely Asp327 and Phe330 (Kim et al., 2006; Maric et al., 2011; Tretter et al., 2011). Therefore, a conformational change at this position would influence collybistin recruitment, thus affecting the efficiency of gephyrin synaptic targeting, and perhaps the ability of gephyrin to immobilize GABA_A_Rs. Pin1 come into play once proline-directed phophorylation has occurred. This molecular switch is therefore positioned downstream the signaling cascades that orchestrate the precise phosphorylation patterns on their corresponding target molecules, thus being able to tune GABAergic transmission.

## Concluding remarks

The different roles played by the scaffolding molecule gephyrin at GABAergic synapses are still not completely understood. Gephyrin builds a stable scaffold underneath the synaptic plasma membrane to guarantee, over time, the appropriate number of GABA_A_Rs being juxtaposed to pre-synaptic releasing sites. Despite its overall stability, the gephyrin scaffold must ensure rapid changes in its composition to sustain several forms of synaptic plasticity. One mechanism promoting dynamic changes at inhibitory PSD is represented by post-translational modifications, and in particular by reversible phosphorylation of several key components of the PSDs. The fact that phosphorylation plays a key role in regulating synapse re-arrangement is not new, being extensively characterized at the level of neurotransmitter receptors. The novelty consists in having identified new signaling pathways able to affect synaptic strength by acting on the scaffolding molecule itself *via* alterations of its clustering properties. We are still at the beginning of this new challenge but the data obtained so far disclose a complex scenario. Several serine and threonine residues were found phosphorylated on gephyrin isolated from mouse and rat brains, thus indicating that multiple pathways converge on gephyrin, modifying residues that are very close to each other and possibly functionally interconnected. Interestingly some of the phosphorylated sites were also found acetylated *in vivo*, raising the possibility that acetylation exerts and additional level of control by directly modulating gephyrin protein-protein interaction or by competing with specific phosphorylation targets.

Unveiling the hierarchy of each phosphorylation event, their cross-talks and their respective contribution to the functional organization of GABAergic synapses will require not only the identification of all kinases and phosphatases involved, but also an accurate analysis of their impact on various gephyrin activities, and in particular on GABA_A_Rs trafficking and synaptic localization.

## Conflict of interest statement

The authors declare that the research was conducted in the absence of any commercial or financial relationships that could be construed as a potential conflict of interest.
